# Semi-Annual Meeting of the Erie County Medical Society, Held at the Rooms of the Society, Tuesday, June 10th, 1862

**Published:** 1862-07

**Authors:** Leon F. Harvey

**Affiliations:** Sec’y.


ART III.—*Semi-Annual Meeting of the Erie County Medical Society, held at the rooms of the Society, Tuesday, June* 10*th*, 1862.

The members were called to order by the President, Dr. Samo, at 10 .45 A. M.

Present—Drs. Samo, C. W. Harvey, Congar, Miner, Gay, Nichell, Ring, Whitney, Shaw, Allen, Dorland, J. R. Lothrop, Cronyn, Mixer, Strong, Gould, L. F. Harvey.

Minutes of the last meeting were read and adopted.

Application of John McKennon was presented and referred to a committee of three, consisting of Drs. Whitney, Ring and Nichell.

A letter from A. R, Parker, Orator for the day, was read, and his excuse for not being able to peform his duty was accepted.

Committee on credentials of John McKennon reported in favor of admitting him a member.

The President reported that he had made out a license for Leroy Parker, who had been examined, but had failed to call for it.

It being moved and seconded that a committee be appointed to appoint an Orator for the next meeting; it was carried. Committee appointed by the President consisted of Drs. Congar, Ring and Whitney.

Dr. Gay moved that Dr. Eastman be invited to read his Address prepared for January meeting to day, if convenient, if not, at some future meeting. Carried.

Dr. Samo read letters from Dr. Hayward in regard to the Sydenham publications, of which, two books, the Society have not received.

Moved and seconded that the Librarian correspond with Dr. Hayward, and request him to furnish ten receipts, and furnish the missing books, and if he fails to obtain satisfaction to write to the Secretary in London.—Carried.

Dr. Whitney moved that the Treasurer at the next meeting make a report of members who have, and have not, paid their bills. Carried.

Committee on Orator, report, that they appoint Dr. Abbott as Orator, and P. S. Dorland as substitute.

Voted to adjourn.

LEON F. HARVEY, Sec'y.

---

THE MEDICAL REFORM BILL, recently passed by Congress, provides for a sanitary inspection by which the army is placed under the constant surveilance of a Medical Bureau. 2, increasing the rank of the Surgeon-General to that of a Brigadier-General. 3, selection of the highest officers according to merit, and not according to seniority.

